# Physiological and Immunological Status of Adult Honeybees (*Apis mellifera*) Fed Sugar Syrup Supplemented with Pentadecapeptide BPC 157

**DOI:** 10.3390/biology10090891

**Published:** 2021-09-09

**Authors:** Ivana Tlak Gajger, Maja Ivana Smodiš Škerl, Petra Šoštarić, Jelena Šuran, Predrag Sikirić, Josipa Vlainić

**Affiliations:** 1 Department for Biology and Pathology of Fish and Bees, Faculty of Veterinary Medicine, University of Zagreb, 10000 Zagreb, Croatia; 2 Agricultural Institute of Slovenia, 1000 Ljubljana, Slovenia; Maja.Smodis.Skerl@kis.si; 3 Department for Pharmacology, Medical Faculty, University of Zagreb, 10000 Zagreb, Croatia; petra.sostaric@mef.hr (P.Š.); predrag.sikiric@mef.hr (P.S.); 4 Department of Pharmacology and Toxicology, Faculty of Veterinary Medicine, University of Zagreb, Heinzelova 55, 10000 Zagreb, Croatia; jsuran@vef.hr; 5 Laboratory for Advanced Genomics, Division of Molecular Medicine, Institute Ruđer Bošković, 10000 Zagreb, Croatia

**Keywords:** honeybee colonies, *Apis mellifera*, pentadecapeptide BPC 157, laboratory-controlled conditions, hemolymph, physiology and immunology parameters

## Abstract

**Simple Summary:**

Administration of BPC 157 induces positive physiological changes in honeybees. Those changes include few biochemical and immunological parameters in hemolymph and hypopharyngeal gland of newly emerged worker bees in laboratory-controlled conditions and increased enzymatic activity in the digestive system of young honeybees in apiary. Changes in proteolytic enzyme activity are particularly visible in the top of the epithelial cells in the mid-guts of honeybees, indicating a connection between oral administration of sucrose diet enriched with BPC 157 and honeybees’ immunity. These cognitions give a firm basis for further studies of BPC 157 use in all aspects of beekeeping.

**Abstract:**

Various factors contribute to a decline in diversity and number of bees. Here, an integrated approach in experimental BPC 157 therapy was implemented, combining laboratory-controlled and field study results. The aim of a study was to assess the effects of BPC 157 additional feeding of newly emerged worker honeybees on few biochemical and immunological parameters in hemolymph (glucose, trehalose, lipids, proteins, vitellogenin, glucose-oxidase (GOX)), and hypopharyngeal gland (HPG), in laboratory-controlled conditions. Additionally, to examine the physiological status of protein digestion, the enzymatic activity of leucine aminopeptidase (LAP) in the mid-guts of worker honeybees was analyzed. It was found that individual honeybees, in hoarding cages, following BPC 157 administration through carbohydrate food, showed positive physiological changes when compared to the control groups. Those results were complemented by strong and visible LAP activity, particularly noticeable in the apical parts of the epithelial cells in the mid-guts of young worker honeybees originated from treated hives, suggesting a link between alternative oral therapy with BPC 157 and honeybees’ immunity.

## 1. Introduction

In a previous study, we reported about the first appliance of stable gastric pentadecapeptide BPC 157 to honeybee colonies with achieved significant therapeutic effects against Nosemosis type C [1], caused by microsporidium *Nosema ceranae* [2]. We hypothesized and confirmed that BPC 157, as a new way of honeybee therapy, can be used to counteract *N. ceranae* invasions in field conditions and increase colonies strength [1]. The role of this microsporidium in honeybee losses or global insects decline remains controversial [3,4] but decreased colony strength, impaired digestion, absorption of nutrients, and shortened lifespan due to energetic stress of individual adult honeybees as well as colony collapse have been reported [5,6].

According to 470/2009/CE [7] and 37/2010/CE European Union regulations [8], the use of antibiotics in beekeeping management practices is not allowed. Currently there is not any registered or authorized veterinary medicine product available for the honeybee diseases control, except acaricides against *Varroa destructor* parasitic mites [9,10]. In the recent decade, an alarming decline of beneficial insects, including honeybees, has been reported at global scale [11,12]. Especially honeybee colonies which represent economically most important social insect pollinators are affected by numerous negative environmental factors [13]. Therefore, a negative influence of different pressures (e.g., parasites, pathogens, pesticides, lack of natural food, unfavorable weather conditions) on honeybee colonies strength, probability of winter survival, and losses have driven the need to develop sustainable alternative therapies. Due to mentioned multifactorial problem in beekeeping sector, the knowledge about physiological and immunological status of honeybees is crucial in advising beekeepers how to counteract colony health alterations and implement good production, veterinary, and environmental management practices [14].

The colony’s strength and survival depend on the social and individual immunity of honeybees. Social immunity is based on honeybee behavioral cooperation to protect the colony against pathogens, such as hygienic behavior or thermoregulatory activity, and on the exchange in defense substances, such as antimicrobial peptide defensin-1 and antiseptic enzyme glucose oxidase (GOX) [15,16]. Individual immunity of honeybees is based on innate immune response, which consists of cellular (phagocytes and hemocytes) and humoral effectors including antimicrobial peptides and various proteins (e.g., vitellogenin). These effectors are also shared between members of the social colony to improve its resistance to pathogens. Components of individual immunity are adaptable in social contact, which means that the level of effectors will change with the colony strength [16]. Glucose oxidase activity is a marker of social immunity of honeybee colony as it is catalyzing the oxidation of β-D-glucose to gluconic acid and hydrogen peroxide (H_2_O_2_) [16]. The latter has antiseptic properties for in-hive storied food [17]. Honeybees use nutrients of plant origin as a resource for these effectors, with their gut being the interface of their immunity [18]. As intestines health is essential for individual immunity, food supplements with mid-gut health-promoting activity could contribute to overall honeybee colony strength.

Pentadecapeptide BPC 157 is a gut-healing, anti-ulcer peptide with general organ protective activity [19,20,21,22,23]. It was tested in the therapy of inflammatory bowel disease (PL-10, PLD-116, PL 14736), in clinical phase II, which revealed a very safe and stable profile with no reported toxicities [24]. It can be administered without any carrier, in µg to ng dose ranges and any route of administration (oral/systematically or topically). It is stable and native in human gastric juice more than 24 h and is considered to be a novel mediator of Robert’s cytoprotection [24]. This pentadecapeptide was successfully used in vivo experiments for the treatment of the variously induced ulcerative colitis and acting as stabilizer of cellular junctions at counteracted leaky gut syndrome [20]. It exerts intestines endothelium protection and counteracts gastrointestinal lesions in rats and mice [19]. Of particular importance for the intestine integrity may be the therapy which activate collateral pathways to recovery occlusive syndromes [25,26,27]. The molecular mechanisms of BPC have been studied in numerous studies including altered genes expression, angiogenesis, NO-system, oxidative stress markers, or prostaglandins system [28,29,30,31,32,33,34,35,36].

Due to its beneficial effect on colonies infected with *N. ceranae*, we hypothesized that it would impact some physiological parameters as well as social and individual immunity markers, such as vitellogenin and GOX, when it is given as a food supplement to honeybees. To reliably assess the potential environmental factors, climatic and variable pasture circumstances or other similar parasites and pathogens loads, as well as to prevent false results, an integrated approach combining field and laboratory study results is required. The aim of this study was to evaluate the effects of BPC 157 supplemental feeding of newly emerged adult worker honeybees on several biochemical (glucose, trehalose, lipids) and immunological parameters (total proteins, vitellogenin, GOX) in hemolymph, in laboratory-controlled conditions. Additionally, the diameters of hypopharyngeal gland (HPGs) acini were measured as one of markers of immunological honeybee status. Furthermore, to examine the physiological status of protein digestion, the enzymatic activity of leucine aminopeptidase (LAP) in mid-guts of hive honeybees was analyzed.

## 2. Materials and Methods

### 2.1. Experiments in Laboratory-Controlled Conditions

#### 2.1.1. Design of Trials in Incubators

Plastic round boxes (dimensions: ~8 cm × 6 cm; height × radius) were drilled on the top cover by 80 circular ventilation holes (each ~2 mm wide). Then, two bigger additional holes (each ~12 mm wide) were made, serving as placeholders for feeding pipes. Adult bees used in experiment were taken from five hive frames with sealed older brood taken from five honeybee colonies (*Apis mellifera carnica*). Frames were put in an incubator (I-115CAS, Kambič Laboratorijska oprema, Semič, Slovenia) at 35 °C and 70% RH and were left through the night. Next day, the newly emerged adult honeybees were carefully collected and transferred into previously prepared cages (see above). In each cage were ~60 adult honeybees.

In our experiment, the part of the sequence of human gastric juice protein, called BPC 157 (Gly-Glu-Pro-Pro-Pro-Gly-Lys-Pro-Ala-Asp-Asp-Ala-Gly-Leu-Val, M.W. 1419 Kda) as peptide with 99% (HPLC) purity, which is freely soluble in saline water at pH 7 (Diagen, Ljubljana, Slovenia), was used. Three mg of BPC 157 was melted in 1.5 L of drinking water to obtain a concentration of 2 µg/mL as a stock solution. This solution was stored in a refrigerator at 4 °C. Sucrose sugar and drinking water were mixed 1:1, (*w*/*v*) and heated to 40 °C. The food for honeybees was made using 1 mL of BPC solution and 100 mL of sugar syrup to obtain 100 ng/1 mL of BPC 157.

We had two groups of honeybees. An experimental group of adult honeybees was fed with sugar syrup supplemented with BPC 157 (BPC group) and the control group was fed with pure sugar syrup. All honeybees were fed *ad libitum*. Each cage was equipped with drinking water through additional plastic tube. Each group had five replicates. Food was freshly prepared every second day and food consumption was recorded on daily basis. Dead honeybees were recorded and removed from cages every day.

#### 2.1.2. Collection of Honeybee Hemolymph Samples

On day 11 and day 15, extraction of honeybee hemolymph was performed for analyses of immunological indicators, and on day 13 and day 22 for biochemical ones. Hemolymph was taken from antenna bases with micro capillaries (10 µL), according to method described in Beebook [37], as a pool of 3 to 5 individual honeybees per cage. For different biochemical analyses, the hemolymph was taken separately in Eppendorf tubes or in vials. All samples were stored at −80 °C.

#### 2.1.3. Biochemical Hemolymph Parameters

##### Glucose, Trehalose and Total Lipids Concentrations

The carbohydrates content in hemolymph was determined using a commercially available kit (Glucose Assay Kit, Sigma-Aldrich, MO, USA). The glucose in samples is oxidized to gluconic acid and hydrogen peroxide. Hydrogen peroxide reacts with o-dianisidine, giving a colored product in the presence of peroxidase. Intensity of a sample color was proportional to the glucose concentration. The optical density was measured using the spectrophotometer model UV 1800 (Shimadzu, Marlborough, MA, USA), at 540 nm. The final amount of glucose was calculated using the standard curve and multiplied by the dilution factor.

Following glucose measurements, enzyme trehalose was added to each well and incubated (37 °C, during 24 h). Measurement was performed after centrifugation at 340 nM. Namely, the molecule of trehalose was hydrolyzed to two molecules of D-glucose in a reaction catalyzed by enzyme trehalose [37]. Concentration of trehalose was calculated by reading the proper reaction minus the previously determined glucose concentration in the same honeybee hemolymph sample. The obtained results were multiplied by the molecular weight of trehalose (342.3 g/mol) and divided by two times the molecular weight of glucose (180 g/mol).

The amount of lipids was determined by using the sulpho-phospho-vanillin method [38]. The sample was mixed with 200 µL of sulphuric acid (Sigma Aldrich, Saint Louise, MO, USA) and incubated for 10 min at 100 °C. After quick chilling on ice, 2 mL of vanillin produced by Sigma Aldrich (Saint Louise, MO, USA) as 13 mM in 66.8% phosphoric acid was added. After 30 min of incubation at the room temperature, the optical density was measured at 546 nm using the spectrophotometer (Shimadzu, Marlborough, MA, USA). A standard calibration curve obtained from the serial dilution of oleic acid was used for calculation of the total lipid concentration.

#### 2.1.4. Immunological Parameters

##### Total Proteins and Vitellogenin Concentrations in Hemolymph

The total protein amount was determined using a commercial kit (Bio-Rad, Hercules, CA, USA), according to the Bradford method [39]. To prevent the melanisation, samples were held on ice during the analyses. Optical density was measured at 595 nm using the UV 1800 spectrophotometer (Shimadzu, Marlborough, MA, USA). The amount of total proteins was calculated from a standard calibration curve prepared from bovine serum albumin (Sigma-Aldrich, Saint Louise, MO, USA). Concentration of vitellogenin was determined using a MyBioSource Elisa kit, according to the manufacturer’s instructions (MyBioSource, San Diego, CA, USA). This kit is based on immunosorbency (interactions between the vitellogenin antibody and the vitellogenin antigen) and a colorimetric detection of proteins on membrane called the horseradish peroxidase (HRP) system. The optical density was measured at 450 nm using the spectrophotometer (Shimadzu, Marlborough, MA, USA). A standard calibration curve was used for calculation of vitellogenin level in analyzed hemolymph sample.

##### Hypopharyngeal Gland Acini Diameters

**Figure 1:**
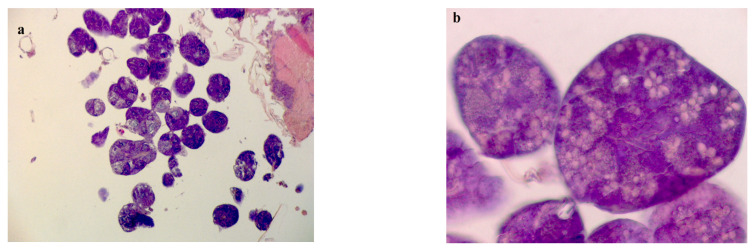
Adult honeybees hypopharyngeal gland tissue microscopic slides used for acinar diameter measuring; (**a**)–10× magnification, (**b**)–40× magnification.

Ten adult honeybees from each cage of experimental BPC 157 and the control group were taken out from plastic experimental cages and ~5 min held in the freezer. Then, the bees HPGs were dissected and prepared for microscopic histological examination using the software Axio Vision 4.6 program (Carl Zeiss, San Diego, CA, USA). Preparation of HPGs tissue included fixation with 10% formaldehyde, dehydration through a series of 70%, 80%, 90%, 100% ethanol solutions (each during 24 h), then 100% 2-propanol (24 h), 100% 2-propanol (12 h), 100% 2-propanol (24 h), 2-propanol: paraffin wax (1:1; 24 h), 3× paraffin wax (each during 24 h), and finally embedded in paraffin wax. Prepared wax blocks were cut using microtome (Leica RM2255, Biosystems Nussloch GmbH, Nussloch, Germany) on 7-µm-thick cuts and placed on microscopic glass slides. Slides were stained with hematoxylin and eosin [40]. Measuring of the HPGs acinar diameters was conducted on 10 to 24 acini (Figure 1) per individual honeybee. Finally, the average value of diameter per group was calculated.

#### 2.1.5. Carbohydrate-Metabolizing Enzymatic Activity

##### Activity of Glucose Oxidase in Honeybee Hemolymph

Activity of glucose oxidase was determined by the horseradish peroxidase/o-dianisidine method using commercially available kit (Sigma Aldrich, Saint Louise, MO, USA). Glucose oxidase catalyzes the oxidation of β-D-glucose to D-gluconolactone and H_2_O_2_ in minute at pH 5.1 and 35 °C. H_2_O_2_ is reduced to water by peroxidase with co-substrate o-dianisidine. With the addition of HCl, the reaction was stopped. The absorption was measured at 400 nm using the spectrophotometer (Tecan, TECAN Group Ltd., Maennedorf, Switzerland). The quantification was performed using H_2_O_2_ as the standard (10–100 μmol/L) with peroxidase and o-dianisidine. The results were expressed as μg H_2_O_2_/h mg protein contained in honeybee hemolymph.

### 2.2. Sampling of Adult Honeybee’s Mid-Guts during Experiments in Field Conditions

#### Leucine Aminopeptidase Enzymatic Activity–Histology Approach

Twenty adult bees were collected from each group during the previously conducted field experiment [1]. Shortly, the additional feeding procedure at apiary was as follows: the experimental group of colonies were fed each day with 0.25 L of sucrose solution mixed with 0.1 μg/mL of BPC 157, and control colonies only with sucrose solution. Additional food was offered in hive feeders, and experimental feeding lasted for 21 consecutive days. The full length of intestines of each individual adult honeybee was pulled out after the short exposure to a low temperature (10 min at 4 °C). For extraction purposes, a larger pair of forceps was used to hold the head and the thorax of each specimen, and a smaller pair of forceps to hold the top of the last abdominal segment where intestines were carefully pulled out. Immediately after, the esophagus and honey sac as well as hind guts including rectum were removed by cutting them off. For examination of LAP enzyme activity, mid-gut samples were fixed in the glass tubes with a cooled (4 °C) solution of formol-calcium during 24 h in the refrigerator. This solution was then discarded, and cooled sucrose was added. Mid-gut samples were stored at 4 °C until further preparation. Degreased cuts of midgut were finally prepared and dyed with special stains for determination and distribution of LAP, according to Hrapchak and Shennan (1980) [41]. All histological preparations were analyzed, as described in previously published studies [42].

The level of LAP enzyme activity was estimated using qualitative and quantitative microscopic examinations. Observations of enzymatic activity were evaluated according to its staining intensity through the adoption of the score ranging from 1 to 4 mark and described as very strong reaction (4), strong reaction (3), moderate reaction (2), and weak-barely detectable reaction (1). Score 4 was given to those highest enzyme activity demand of crucial relevance accordance with the experience of the researcher. In order to avoid bias, all scores were provided without allowing the view of the scores given by other researcher. Microscopic examination was performed under a bright field microscope Olympus Bx41 (Olympus, Tokyo, Japan), at 10× and 40× magnification. The figures record of the microscopic mid-gut preparations was taken using an Olympus DP12 U-TVO camera (Olympus, Tokyo, Japan).

### 2.3. Statistical Analyses

To assess and verify the statistically significant differences in obtained results between the experimental and control group at different sampling occasions, the one-way analysis of variance (ANOVA) with Turkeys’ post-hoc test, for multiple comparisons, and the Mann–Whitney U test were conducted using the statistical software package GraphPad Prism software version 7.00 for Windows (GraphPad Software, La Jolla, CA, USA). Results were checked for normality using the Shapiro–Wilk test. All results are presented as the main values and standard deviations. Statistical significance testing was conducted with a significance level of α = 0.05 to define statistical differences (0.95 confidence interval).

## 3. Results

### 3.1. Consumption of Diets and Survival Rates of Adult Honeybees

**Figure 2:**
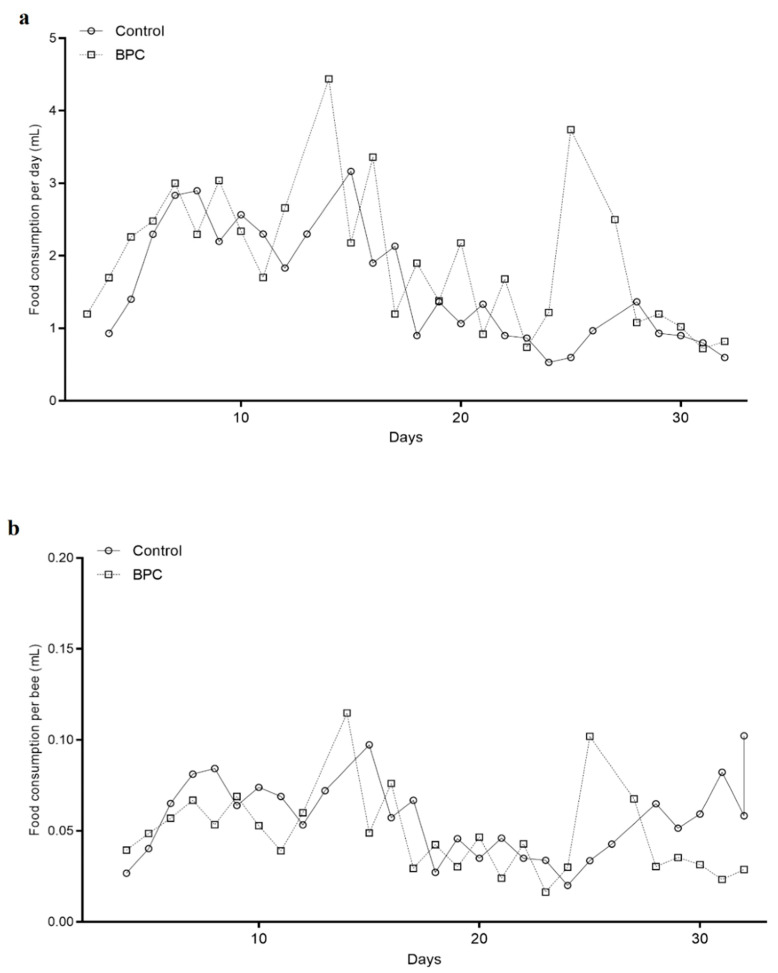
Food consumption amounts during experiments in laboratory-controlled conditions, for the BPC and control group (**a**)–cumulative amounts of diet consumption per day, (**b**)–amounts of diet consumption per bee). No statistically significant differences.

Consumption analyses of honeybees on a daily basis during experiments in laboratory-controlled conditions showed no significant difference in cumulative diet consumption, as well as in amounts of food consumption per individual bee, between the experimental BPC and control groups (Figure 2). The mean cumulative consumption was 1.96 ± 0.96 mL per the experimental BPC group and was 1.52 ± 0.79 mL for the control.

**Figure 3:**
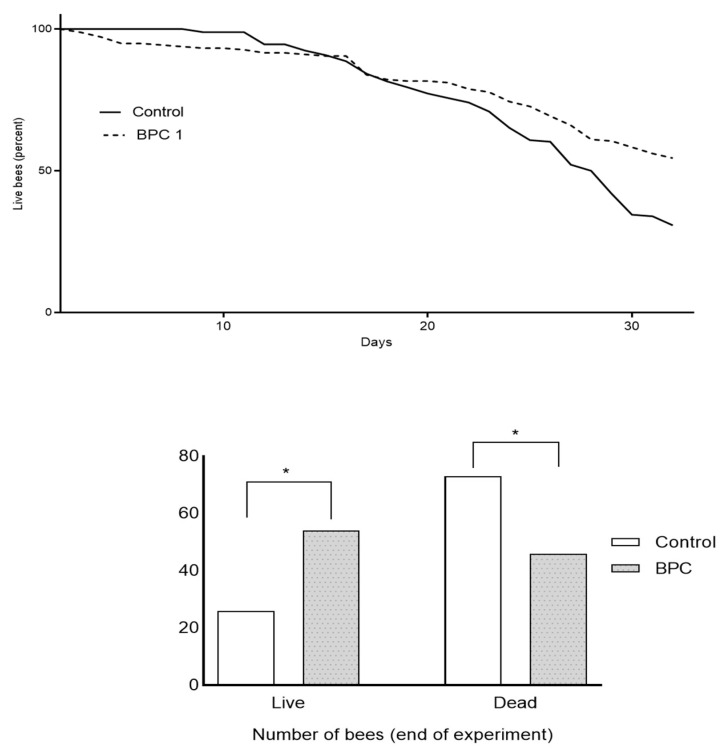
The survival curves of adult honeybees during experiments in laboratory–controlled conditions for the experimental BPC and control group. Asterisks indicate statistically significant differences: BPC vs. control, live/dead adult bees, * *p* < 0.05.

Survival analysis of adult honeybees during and at the end of trials in laboratory-controlled conditions showed significant differences in mortality rates between experimental groups (* *p* < 0.05; df = 4). In BPC, a supplemented honeybee group positive survival trend was observed when compared to those fed with only sugar syrup (Figure 3). At the end of the experiment, differences in mean numbers of live (52.00 ± 11.14 vs. 42.67 ± 1.15) and dead honeybees (48.00 ± 11.4 vs. 57.33 ± 1.15) in mentioned experimental and control groups were observed and reached statistical significance.

### 3.2. Biochemical Hemolymph Parameters of Adult Honeybees Fed with BPC

**Figure 4:**
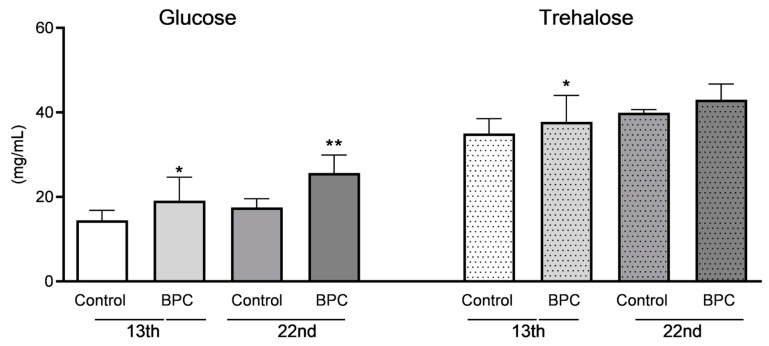
Carbohydrate’s concentrations in adult honeybee hemolymph sampled on the 13th and 22nd day of the experiment under laboratory-controlled conditions for experimental the BPC and control groups. Asterisks indicate statistically significant differences: BPC vs. control, * *p* < 0.001, ** *p* < 0.0001; mean ± SD.

The concentrations of glucose were significantly higher in the hemolymph of adult bees of the experimental group which was fed with sucrose syrup enriched with pentadecapeptide BPC 157, when compared to the control group of bees (Figure 4) (F = 27.59, *p* < 0.001). This difference was observed in hemolymph of 13-day-old adult bees as well as in 22-day-old bees. In detail, glucose concentrations in BPC group were 19.10 ± 5.57 mg/mL on the first hemolymph sampling occasion, and 27.90 ± 5.36 mg/mL on the second. Concentrations of glucose in the control group were relatively similar during the whole experiment period. They varied in range from 14.46 ± 2.38 mg/mL to 19.10 ± 5.54 mg/mL, respectively.

Similarly, concentrations of trehalose were significantly higher in the samples of younger adult bees hemolymph (13 days old) of the experimental BPC group when compared to the control (F = 27.59, *p* < 0.0001). The main trehalose concentrations slowly increased during experiment from 37.80 ± 6.24 mg/mL to 45.51 ± 4.59 mg/mL (Figure 4). A significant difference was observed only at the 13th day of the experiment in the treated and control groups, although tendency to raise trehalose content in hemolymph was at the same level.

**Figure 5:**
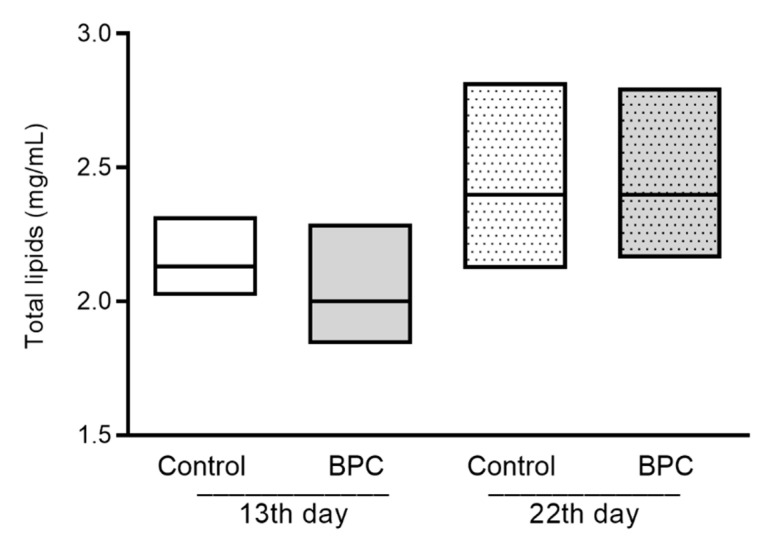
Total lipid concentrations in adult honeybee hemolymph sampled on the 13th and 22nd day of the experiment under laboratory-controlled conditions for the experimental BPC and control groups. No statistically significant differences for BPC vs. control were found.

Total lipid concentrations in hemolymph samples of honeybees in the experimental BPC and control groups varied during the whole observation period, but there were no significant differences found among mentioned groups (F = 5.02, *p* > 0.05). In the first and second sampling occasion, the mean values of lipid concentration in the experimental BPC group were 2.03 ± 0.18 mg/mL and 2.55 ± 0.27 mg/mL, while it varied from 2.15 ± 0.12 mg/mL to 2.43 ± 0.25 mg/mL in the control group (Figure 5).

### 3.3. Effect of Food Supplement BPC 157 on Immunological Parameters

**Figure 6:**
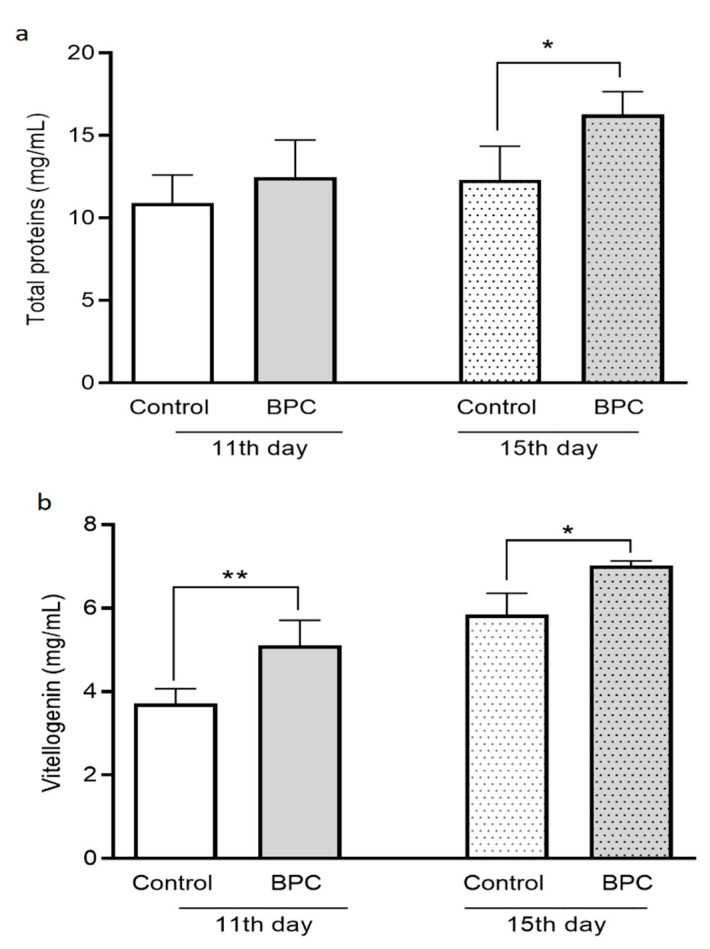
Concentrations of total proteins (**a**) and vitellogenin (**b**) in adult honeybee hemolymph sampled on the 11th and 15th day of the experiment under laboratory-controlled conditions for the experimental BPC and control groups. Asterisks indicate statistically significant differences: BPC vs. control, total proteins * *p* < 0.05, vitellogenin ** *p* < 0.0001, * *p* < 0.05; mean ± SD.

The concentrations of total proteins in samples of adult honeybees were significantly higher just in the BPC group of 15-day-old honeybees in comparison with the concomitant control group (F = 6.0; *p* < 0.05) (Figure 6a). Vitellogenin concentrations were significantly higher in the samples of adult honeybees’ hemolymph in experimental BPC groups (Figure 6b) compared with their pertaining controls (F = 39.74; ** *p* < 0.0001, * *p* < 0.05), in both sampling occasions. In more detail, the mean values of determined vitellogenin concentrations in 11- and 15-day-old honeybees kept under laboratory-controlled conditions were 5.10 ± 0.60 mg/mL and 7.01 ± 0.11 mg/mL for the BPC group; and for pertaining control groups they were as follows: 3.71 ± 0.35 mg/mL and 5.84 ± 0.51 mg/mL, respectively.

**Figure 7:**
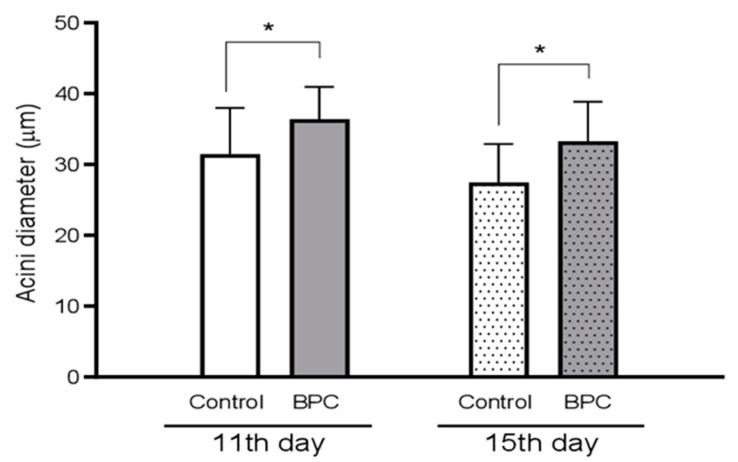
Acini diameter of hypopharyngeal glands dissected from 11- and 15-day-old honeybees, during experiments in laboratory–controlled conditions, for the experimental BPC and control groups. Asterisks indicate statistically significant differences comparing the mean acini diameters between the BPC and control groups, * *p* < 0.01, mean ± SD.

The obtained results regarding acini diameter of HPGs dissected from 11- and 15-day-old honeybees, during experiments in laboratory–controlled conditions, for the experimental BPC and control groups are shown in Figure 7. Hypopharyngeal gland acini diameters from experimental group fed with BPC were significantly larger compared with HPGs acini originated from same age honeybees of the control group fed with pure sugar syrup (df = 42, *p* < 0.001), at both observation dates.

**Figure 8:**
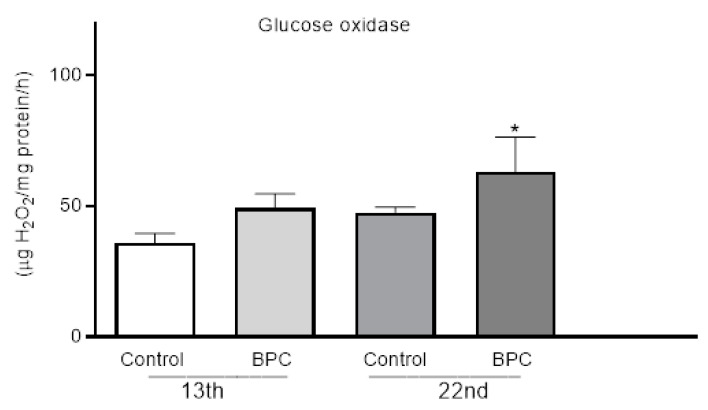
Levels of carbohydrate-metabolizing enzyme glucose oxidase activity in adult honeybee hemolymph sampled on the 13th and 22nd day of the experiment under laboratory-controlled conditions for experimental the BPC and control groups. Asterisks indicate statistically significant differences: BPC vs. Control, * *p* < 0.01, mean ± SD.

The GOX activity level in 22-day-old adult honeybee hemolymph of the experimental BPC group was statistically higher than in the control group (F = 8.21; *p* < 0.01). Determined mean values during the observation period for the BPC group were 49.10 ± 5.57 µg H_2_O_2_/mg protein/h and 62.90 ± 13.48 µg H_2_O_2_/mg protein/h, while, in the hemolymph of adult honeybees of same age originated from the control, they were 35.71 ± 3.80 µg H_2_O_2_/mg protein/h and 47.50 ± 2.0 µg H_2_O_2_/mg protein/h, respectively. Results are presented in Figure 8.

### 3.4. Leucine Aminopeptidase Activity in Mid-Gut of Bees Fed with BPC 157

**Figure 9:**
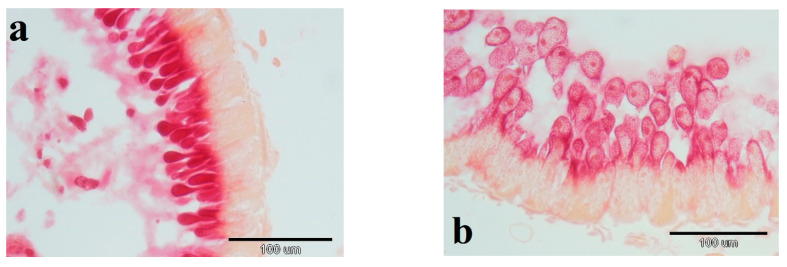
Leucine aminopeptidase enzymatic activity in mid-gut enterocytes of adult honeybee originated from colony previously fed sugar syrup supplemented with BPC 157 (**a**), and untreated colony fed only sugar syrup (**b**); magnification ×40.

In microscopic preparations of midgut samples of honeybees taken from the experimental BPC group, strong and apparent LAP activity was determined (scores 4/4), which was particularly noticeable in the apical parts of the epithelial cells. In the mid-guts of honeybees taken from the control group, LAP activity was barely visible (scores 1-2/4) in the partially destroyed epithelium layer (Figure 9). Microscopy assessment of wall layers and epithelial cells in mid-guts showed a mostly regenerated epithelium layer of mid-guts originated from honeybees previously fed sucrose syrup enriched with BPC 157.

## 4. Discussion

A previously published study, BPC 157, showed significant therapeutic effects on reducing the development of the microsporidium *N. ceranae* spores, regeneration of mid-gut epithelial layer, and significantly stronger colonies at the end of field trial [1]. In general, BPC 157 is highly effective in the therapy of several diseases and conditions in different species, and it is safe for clinical trials for review, see [21,22]. In addition, BPC 157 is primarily a healing peptide known to modulate the expression of genes involved in wound healing mechanisms [34]. Due to this, it seems to be suited to achieve a desirable counteraction in therapy, and we hypothesized that it would exert a similar immunomodulatory action in honeybees. The most convenient application to study its impact on honeybee physiology was when given as a food supplement.

In order to avoid apian-based production with risk for the accumulation of residues in beehive products, used as food for humans, beekeepers are extremely interested in using only alternative management practices with substances of natural origin in combination with api-bio-technical actions as preventive measures for keeping colonies healthy [43].

In our study, we tested whether the feeding with sugar syrup supplemented with BPC 157 can shape baseline immunocompetence by measuring physiological and immunity parameters in hemolymph, HPG, and activity of LAP in mid-guts of honeybees. As environmental factors are often variable and honeybee colonies have specific life manners, we combined experimental feeding of honeybees with known number and age in laboratory-controlled conditions. Furthermore, we analyzed the proteolytic enzyme activity in mid-guts from honeybees sampled from hives in the experimental apiary.

Opposite to studies that reported that honeybees ate significantly higher amounts of food supplemented with micronutrients [44], in the current study, honeybees from both groups consumed similar amounts of offered food. A similar observation was reported after exposition to probiotic treatments [45]. Recently, interesting results were published regarding worker bees ingestion of lipids in almost optimal amount in relation to the protein content in the offered diet [46].

The survival curves of adult bees during experiments in laboratory-controlled conditions indicate a positive survival trend for the BPC 157 supplemented honeybee group in comparison with the control group. This is in accordance with the absence of negative impacts on health and improved strength of honeybee colonies during additional feeding with BPC 157 in apiary [1] and confirms the safety of BPC 157 for clinical trials in field and laboratory conditions.

Concentrations of glucose for both hemolymph sampling occasions, and trehalose for first sampling were significantly higher in the BPC 157 experimental groups. Cariveau and collaborators (2014) [47] suggested that beneficial microorganisms could more efficiently colonize intestines if they contain sugar-rich medium because of diverse sugar degradation pathways. Regarding this, previously published results have confirmed that food supplements containing *Lactobacillus* spp. and *Bifidobacterium* spp. or their metabolites improve the defense against pathogens [45,48,49] as well as carbohydrate up-take and utilization, including biosynthesis of trehalose which is used as energy storage in bees [50]. In this study, BPC 157 increased the levels of sugars in honeybee hemolymph, while, in the previous, it increased the resistance to the pathogen *N. ceranae*. This could imply that the immune response occurs immediately at the gut level. The potential interaction of BPC 157 with the microbiome contributes to gut homeostasis and more efficient digestion.

Total lipid concentrations were stable during the whole experiment with no significant differences between the experimental and control groups, which can be explained in respect to sugar syrup-based diets of caged honeybees in laboratory-controlled conditions. Although, these results were different from earlier reported [50].

Concentrations of total proteins were significantly higher in the hemolymph of honeybees that originated from the experimental BPC 157 group, sampled in second occasion (15-day-old honeybees), in comparison with the control group, and are in accordance with earlier conducted studies regarding impacts of sterols on various insects’ fitness traits [41]. Vitellogenin has an important role in workers division of labor, queen longevity, and reproduction, as well as protection to oxidative stress [51]. Except in the queen, it is possible to detect vitellogenin in hemolymph of workers [52], and its concentrations can be changed similar to a response to diet, so it is also a marker of nutritional [53] and immunity status due to an ability to bind to multiple pathogens [54,55]. Furthermore, worker bees that fed a royal jelly have increased vitellogenin expression in the ovaries, but it has non-reproductive functions such as regulation of behavior patterns [56]. In our study, vitellogenin concentrations in adult honeybee hemolymph sampled on the 11th and 15th day of the experiment under laboratory-controlled conditions showed a significant increase in comparison with honeybees fed only sugar syrup. These results encourage the possibility that BPC 157 could stimulate the immune honeybee’s response after proper and regular usage at apiary level. Additionally, they are in accordance with the results of Evans and Lopez (2004) [57].

Furthermore, in this study, we observed a significant increase in HPGs acini diameters in younger honeybees, originated from the BPC 157 group, which can be predicted as possibly prolonged in-hive function as nurse workers due to positive physiological reaction on food supplementation. Although the increased size of HPG acini does not always mean good nutritional status [58,59], here it is combined with the significant increase in vitellogenin concentrations. This antioxidative protein is linked with honeybee longevity, which can be linked with the positive survival trend of honeybees fed with BPC 157.

It is known that honeybees have only one third numbers of genes responsible for immune reactions, in comparison with other insects with solitary behavior [60]. That means that honeybee colonies must also possess other types of defense mechanisms against pathogens, e.g., GOX, which provide honeybees a better in-hive antiseptic protection [17]. Here, we found that the activity of GOX in adult honeybee hemolymph sampled on the 22nd day of the experiment was significantly higher in the BPC 157 fed group than in its pertaining control group. This finding suggests that BPC 157 food supplementation is welcome for development and maintenance of normal honeybees’ immunological functions. By increasing levels of GOX, BPC 157 promotes the social immunity of honeybees.

Diverse natural protein food is required for honeybee’s body tissues development and sustains immunocompetence. The consumption of adequate provisions of polyfloral ensures the intake of specific proteins and essential amino acids in good rations [61]. To examine the physiological status of protein digestion in mid-guts of individual adult bees from colonies located in apiary, we decided to estimate the activity of LAP proteolytic enzyme. Significantly stronger LAP activity was found in apical parts of the epithelial mid-gut cells sampled from bees fed with BPC 157. This assumption is in accordance with earlier reported study which showed that LAP activity decreases with level of *Nosema* spp. invasion [62], which causes immunosuppression and lower digestion efficiency [63]. BPC 157 could induce the production and secretion of LAP, consequently and indirectly improving the proteins’ digestion and nutrients’ absorption.

All these new cognitions give a good basis for further studies of BPC 157 use in all aspects of beekeeping. Additionally, it is interestingly that, in plants, LAP is a product of octadecanoid pathway and is involved in late wound response [64,65] as an essential defense mechanism. Since BPC 157 promotes wound healing via gene expression modulation in animals, notably Egr, Nos, Srf, Vegr, Akt1, Plcɣ and Kras genes [33], it would be interesting to study its efficacy in plants and the potential activation of LAP genes (LapA1 and LapA) known to be involved in wound-healing response in plants [66].

## 5. Conclusions

According to obtained results, it seems that the administration of sucrose-based diets enriched with BPC 157 can stimulate the functioning of physiological processes and improve immunological status of fed honeybees. This practice can be proposed as a measure for the prevention of visible manifestation of conditional diseases at apiary conditions, and as adjuvant therapy in the anti-varroa management strategies. Finally, this is the second study reporting the newly obtained beneficial cognitions about the BPC 157 oral administration to social insects.

## Data Availability

The data presented in this study are available on request from the corresponding author.
